# Antidepressant Misuse in Esports: A Review of Pharmacology, Adverse Effects, Pharmacokinetics and Biomatrix‐Based Detection for Anti‐Doping Applications

**DOI:** 10.1002/dta.70131

**Published:** 2026-08-11

**Authors:** Kassim Ali Salum, Shao‐Hsin Hung, Meng‐Tsang Hsieh, Mei‐Chich Hsu, William Chih‐Wei Chang

**Affiliations:** ^1^ Doctoral Degree Program in Toxicology, College of Pharmacy Kaohsiung Medical University Kaohsiung City Taiwan; ^2^ Department of Natural Science The State University of Zanzibar Zanzibar Tanzania; ^3^ Department of Neurology Chi Mei Medical Center Tainan City Taiwan; ^4^ School of Medicine, College of Medicine National Sun Yat‐sen University Kaohsiung City Taiwan; ^5^ Department of Sports Medicine, College of Medicine Kaohsiung Medical University Kaohsiung City Taiwan; ^6^ School of Pharmacy, College of Pharmacy Kaohsiung Medical University Kaohsiung City Taiwan

**Keywords:** cognitive enhancement, doping, Esports Integrity Commission (ESIC), governance, World Anti‐Doping Agency (WADA)

## Abstract

Esports has undergone rapid expansion over the past two decades, bringing with it challenges analogous to those observed in traditional athletic domains, particularly the misuse of performance‐enhancing drugs (PEDs). Doping poses a dual threat to competitive integrity and player health, yet its manifestation in Esports remains insufficiently examined compared with conventional sports. This disparity has contributed to the under‐recognition of substance misuse in professional gaming and the emergence of associated health risks among players. A lack of awareness among tournament organisers and regulatory bodies has further impeded the establishment of comprehensive anti‐doping frameworks. To address this gap, the Esports Integrity Commission (ESIC) introduced a prohibited substances list tailored to the cognitive and psychological demands of Esports, acknowledging the limitations of the World Anti‐Doping Agency (WADA) framework in this context. Substances commonly implicated in Esports doping include nootropics and other cognitive enhancers that improve attention, reaction speed and mental stamina. While stimulants such as medications for attention‐deficit/hyperactivity disorder (ADHD) and energy drinks have been widely discussed, the non‐medical use of antidepressants remains underexplored and insufficiently regulated despite their inclusion on ESIC monitoring lists. This review provides a critical synthesis of the pharmacology, pharmacokinetics, adverse effects and analytical detection strategies for antidepressants relevant to Esports doping. A co‐citation analysis of 3756 references published between 2000 and 2025 was conducted to map the field's intellectual structure. Systematic screening identified 69 key studies that met predefined inclusion criteria and were analysed in detail.

## Graphical abstract

This review highlights the misuse of antidepressants as potential cognitive‐enhancing agents in Esports. Despite pharmacological effects that overlap with those of psychostimulants, antidepressants remain an under‐recognised source of doping in Esports. The review also summarises suitable biological matrices, detection strategies, and analytical approaches for antidepressant monitoring in Esports anti‐doping.

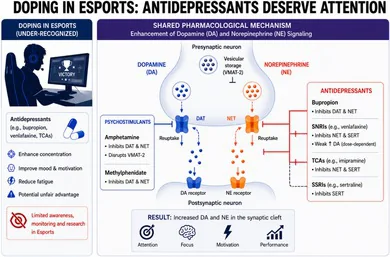

## 1 Introduction

Esports (electronic sports) constitute a professionalised segment of the gaming industry centred on organised, competitive video game play designed to engage audiences, foster rivalry and deliver high‐production entertainment [1, 2]. Esports have existed since 1972, when 24 students competed in what is considered the first recorded Esports tournament. However, it was not until the late 1990s that organised gaming leagues and tournaments offering substantial prize pools were established [3]. Today, Esports constitutes a global cultural and economic phenomenon, attracting millions of spectators, generating substantial sponsorship revenues and establishing itself as a major sector within the broader entertainment and sports industries [4]. Global Esports revenue is projected to reach approximately $12.10 billion by 2030 [5].

This rapid expansion has intensified the competitive pressures faced by professional Esports players, both in high‐stakes tournament environments and during rigorous training sessions, which can lead some of them to encounter depression and anxiety. Evidence supporting this concern comes from a Brazilian study comparing mental health symptoms between Esports athletes and traditional sports athletes, which reported a higher prevalence of depressive symptoms among Esports players [6]. One potential contributing factor is the limited level of physical activity inherent to Esports, as regular exercise is known to alleviate symptoms of depression and anxiety. While this characteristic may lower the barrier to entry for Esports participation, it may simultaneously increase vulnerability to mental health disorders. In addition, prolonged screen exposure—an intrinsic component of Esports training and competition—has been associated with the exacerbation of depressive and anxiety symptoms. Extensive daily screen time may also disrupt sleep patterns, leading to sleep dysregulation, which, in turn, contributes to poorer mental health outcomes [7]. Furthermore, recent studies report that over 80% of professional Esports athletes experience major mental health challenges, such as depression, anxiety and burnout, due to intense training and dense competition schedules [8, 9]. These demands may encourage players to neglect their physical and mental well‐being in pursuit of performance.

These findings suggest a potentially higher risk of antidepressant misuse among Esports players. In response to growing concerns over doping and competitive integrity, the ESIC was established in 2016 as the first non‐profit organisation dedicated to combating cheating in Esports. ESIC recognised that reliance on the WADA code alone does not adequately reflect the unique cognitive demands and competitive characteristics of Esports [10]. Consequently, ESIC introduced a new monitoring list that includes several antidepressants not currently prohibited under the WADA framework [11], allowing for the collection of epidemiological data on patterns of use before potential prohibition.

Currently, the misuse of nootropics such as amphetamine in Esports is well documented, as evidenced by numerous scientific publications addressing the risks associated with their use [12, 13, 14]. However, significant knowledge gaps remain regarding the misuse of antidepressants in Esports, particularly with respect to their pharmacology, pharmacokinetics, adverse effects and analytical detection. As Esports continues to progress towards inclusion in major international sporting events, including the 2026 Asian Games and the 2027 Esports Olympic Games, concerns related to doping practices and player health have become increasingly urgent. Therefore, the primary aim of this study is to evaluate the toxicological relevance, potential health risks and analytical detection associated with antidepressant misuse in Esports, to support evidence‐based anti‐doping frameworks, safeguard player health, preserve the integrity and fairness of competition and inform future monitoring and regulatory decisions consistent with WADA and ESIC principles.

## 2 Materials and Methods

### 2.1 Eligibility Criteria

To achieve the objectives of this review, we examined studies addressing doping in Esports, mental health effects associated with antidepressant misuse, the pharmacokinetics of antidepressants in humans and analytical methods for quantifying antidepressants in biological matrices using liquid chromatography. Particular emphasis was placed on studies employing tandem mass spectrometry (MS/MS) or higher order mass spectrometry (MS^n^) for detection. LC–MS/MS was selected because it enables the direct analysis of antidepressants without the need for derivatisation, making it particularly suitable for compounds with diverse physicochemical properties. This technique offers several advantages, including high sensitivity and selectivity, excellent quantitative performance, broad applicability to polar, non‐polar and thermally labile compounds and simpler sample preparation, thereby reducing overall analytical time and improving laboratory throughput. Although several analytical methods have successfully employed gas chromatography coupled with mass spectrometry (GC–MS) for the determination of antidepressants [15, 16], GC–MS analysis often requires an additional derivatisation step [17], because many antidepressants are non‐volatile, making them unsuitable for direct GC analysis. Derivatisation enhances analyte volatility and thermal stability; however, it increases the complexity of the analytical workflow, prolongs sample preparation, increases reagent consumption and cost and may introduce additional analytical variability.

These advantages make LC–MS/MS particularly well suited for anti‐doping laboratories, where rapid turnaround, high analytical sensitivity and robust quantitative performance are essential. Therefore, LC–MS/MS represents a more practical and efficient analytical platform for the determination of antidepressants in oral fluid.

Articles involving in vitro investigations or analytical techniques other than LC–MS/MS were excluded. Additionally, only English‐language publications from 2000 to 2025 were considered.

### 2.2 Search Strategy

A review with a systematic literature search was conducted using the databases of ScienceDirect, PubMed and Google Scholar. The search strategy incorporated keywords and controlled terms, used individually and in combination, including ‘doping’, ‘Esports’, ‘antidepressants’, ‘drug abuse’, ‘mental illness’, ‘drug testing’, ‘anti‐doping’, ‘liquid chromatography–mass spectrometry’, ‘depression’, ‘oral fluid’, ‘urine’, ‘blood’ and ‘sample extraction’, ‘pharmacokinetics’, ‘excretion profile’ and ‘pharmacology’. Retrieved records were screened at the title and abstract levels to assess eligibility, followed by full‐text review of studies meeting the inclusion criteria. Reference lists of all eligible articles were manually examined to identify additional relevant publications. Supplementary information not available in peer‐reviewed literature was obtained from authoritative websites and professional blogs and was used solely to provide contextual background. Following screening and eligibility assessment, a total of 69 papers were deemed relevant and included in this review.

## 3 Results and Discussion

### 3.1 Doping in Esports

Esports represents a hybrid domain combining digital and physical demands, within which doping can manifest in both real‐world and virtual forms. In the real world of Esports, players have been reported to misuse prescription central nervous system (CNS) stimulants, including amphetamine, methylphenidate and lisdexamfetamine, which are marketed under brand names such as Adderall, Ritalin and Vyvanse, respectively. These substances increase dopamine and norepinephrine levels in the brain, thereby enhancing focus, concentration, reaction time and wakefulness [18]. These substances may confer a competitive advantage, particularly in fast‐paced genres such as first‐person shooter (FPS) games. Public disclosures, including the widely cited statement by professional player Korey ‘Sephis’ Friesen—‘We are all on Adderall … I don't even care’ [19], underscore the prevalence and normalisation of stimulant use within the Esports community. In the virtual world, Esports competitors may resort to software or hardware modifications that provide artificial advantages referred to as e‐doping [14, 18, 20]. The present study focuses primarily on pharmacological doping rather than digital cheating mechanisms. Compared with traditional sports, where doping scandals involving athletes such as Lance Armstrong [21], Diego Maradona [22] and Maria Sharapova [23] have received extensive public and regulatory scrutiny, doping in Esports remains relatively unnoticed and under‐regulated [12, 24]. This regulatory gap creates opportunities for the misuse of prescription medications, including antidepressants, among Esports players. According to WADA, a substance may be included on the Prohibited List if it meets at least two of the following three criteria: (a) It has the potential to enhance, or does enhance, sport performance; (b) it represents an actual or potential health risk to the athlete; and (c) it violates the spirit of sport [25]. In the context of Esports, the non‐therapeutic misuse of antidepressants may satisfy all three criteria. Such misuse has the potential to enhance cognitive performance, may pose health risks when used without a legitimate medical indication or a valid Therapeutic Use Exemption (TUE) and may undermine the spirit of sport by compromising the principles of ethics, honesty, fair play and respect. Consequently, the non‐therapeutic misuse of antidepressants may meet the WADA criteria for inclusion on the Prohibited List.

### 3.2 Statistics of Antidepressant Misuse in Esports

Survey‐based evidence indicates that pharmacological performance enhancement may occur in Esports. Ip et al. reported that, among prescription medications used to enhance Esports performance (*n* = 32), atomoxetine accounted for one response (3.1%), providing evidence of misuse for cognitive or performance enhancement in Esports contexts [13]. Atomoxetine is commonly prescribed for attention‐related disorders and is pharmacologically related to medications used in the management of mood disorders. Consistent with the broader clinical role of such medications, Allen noted that antidepressant treatment during the acute phase of major depressive episodes aims to achieve symptom remission and restore patients to their baseline level of functioning [26]. This therapeutic framework may also apply to Esports players who experience depressive symptoms while engaged in high‐pressure competitive environments. Furthermore, Han and Renshaw reported that, following an eight‐week medication trial, participants receiving Bupropion demonstrated greater reductions in depressive symptoms and significant improvements in online gaming addiction severity compared with those receiving a placebo. These findings suggest that bupropion may alleviate depressive mood while simultaneously reducing excessive gameplay among individuals with comorbid moderate depressive disorder and online gaming addiction [27]. Although antidepressant and anxiolytic medications may be clinically indicated under appropriate medical supervision, their affect‐regulating properties may also increase the potential for non‐medical use or misuse in competitive Esports environments. In performance‐driven settings, players may self‐administer such medications not only to manage psychological distress but also to maintain emotional stability, focus and endurance during training and competition, thereby blurring the boundary between therapeutic use and performance enhancement. Testimonies from former professional players have further highlighted the normalisation of psychoactive substance use in competitive titles such as Counter‐Strike: Global Offensive, Dota 2, Overwatch, Call of Duty, Halo and Fortnite [28].

Despite increasing recognition of cognitive doping in Esports, regulatory oversight of antidepressant misuse remains fragmented and insufficiently developed [29, 30]. Current anti‐doping efforts primarily focus on well‐established psychostimulants, such as amphetamine and methylphenidate, which the ESIC prohibits [11], whereas antidepressants with overlapping pharmacological mechanisms are largely subject to monitoring rather than formal prohibition. Similarly, the IESF aligns its anti‐doping framework with the WADA Code, which primarily targets substances with established performance‐enhancing potential, including amphetamine and methylphenidate [31]. Consequently, the misuse of antidepressants with potential cognitive‐enhancing effects remains an emerging regulatory challenge.

Compared with traditional sports, Esports currently lacks harmonised testing protocols, standardised Therapeutic Use Exemption (TUE) procedures and validated analytical strategies for monitoring emerging psychoactive substances. This regulatory gap not only increases the risk of non‐therapeutic misuse of prescription antidepressants but also complicates the distinction between legitimate clinical treatment and intentional performance enhancement. These challenges highlight the need for Esports‐specific anti‐doping policies, risk‐based monitoring programmes and analytically robust detection methods to safeguard player health, preserve competitive integrity and further harmonise Esports governance with established anti‐doping principles.

### 3.3 Similarities of the Pharmacology of Antidepressants and Psychostimulants

Amphetamine and methylphenidate, with their derivatives, are commonly prescribed for the treatment of ADHD [32]. These substances enhance cognitive performance by increasing dopaminergic and noradrenergic neurotransmission within the central nervous system, resulting in improved alertness, attention, executive function, reaction time and reduced fatigue [33, 34]. Importantly, these neurochemical pathways are also targeted by several classes of antidepressants, particularly tricyclic antidepressants (TCAs), serotonin–norepinephrine reuptake inhibitors (SNRIs) and atypical antidepressants such as bupropion [33, 35].

Although psychostimulants are characterised by a more rapid onset of action and a greater abuse potential [36] than antidepressants [37], both drug classes increase synaptic monoamine availability through modulation of dopamine, norepinephrine and/or serotonin neurotransmission [38, 39, 40]. Amphetamine promotes monoamine release, inhibits monoamine transporters, interferes with vesicular monoamine transporter‐2 (VMAT‐2) and partially inhibits monoamine oxidase, whereas methylphenidate primarily inhibits dopamine and norepinephrine transporters [33]. Likewise, many antidepressants exert their therapeutic effects by inhibiting the reuptake of one or more monoamine neurotransmitters, thereby enhancing synaptic neurotransmission involved in attention, cognitive flexibility, emotional regulation, reaction time and fine motor coordination—functions that are particularly relevant to competitive Esports performance. The pharmacological mechanisms of ESIC‐prohibited psychostimulants and the antidepressants included in the ESIC Monitoring Program investigated in this study are presented in Table 1.

**TABLE 1:** Pharmacological similarities between psychostimulants prohibited by the Esports Integrity Commission (ESIC) and antidepressants included in the present study.

Drug/class	Representative drugs	Pharmacological target	Neurotransmitters affected	Potential relevance to Esports performance
Psychostimulants (ESIC prohibited)	Amphetamine, methylphenidate	DAT, VMAT‐2 NET	Dopamine ↑↑, Norepinephrine ↑↑	Increased alertness, attention, vigilance, reaction time, and reduced fatigue
Tricyclic antidepressants (TCAs)	Amitriptyline, imipramine, nortriptyline, desipramine	SERT and NET inhibition [41, 42]	Serotonin ↑, norepinephrine ↑ (nortriptyline/desipramine predominantly NET) [43, 44, 45]	Enhanced concentration, cognitive processing, mood regulation
Selective serotonin reuptake inhibitors (SSRIs)	Escitalopram, sertraline	Selective SERT inhibition [46]	Serotonin ↑ [47]	Improved emotional regulation, reduced anxiety and stress that may indirectly influence performance
Serotonin–norepinephrine reuptake inhibitors (SNRIs)	Venlafaxine	SERT and NET inhibition (dose‐dependent)	Serotonin ↑, norepinephrine ↑ [48, 49], weak dopamine ↑ [50, 51]	Improved sustained attention and cognitive function through enhanced monoaminergic signalling
Atypical antidepressants	Bupropion	DAT and NET inhibition	Dopamine ↑, norepinephrine ↑ [52]	Pharmacologically resembles psychostimulants and may improve alertness and concentration

Collectively, these pharmacological similarities provide a mechanistic basis for considering certain antidepressants within the context of anti‐doping in Esports. While antidepressants are legitimate therapeutic agents when prescribed appropriately, several members of this drug class share neurochemical mechanisms with prohibited psychostimulants, particularly through modulation of dopaminergic and noradrenergic signalling (Figure 1). Consequently, their non‐therapeutic misuse may confer cognitive advantages relevant to Esports performance, including enhanced attention, sustained vigilance, emotional regulation, reaction speed and fine motor control. These observations support continued surveillance and evidence‐based evaluation of antidepressant misuse within anti‐doping frameworks, with the aim of protecting player health, preserving competitive fairness and maintaining the integrity of Esports competitions.

**FIGURE 1:**
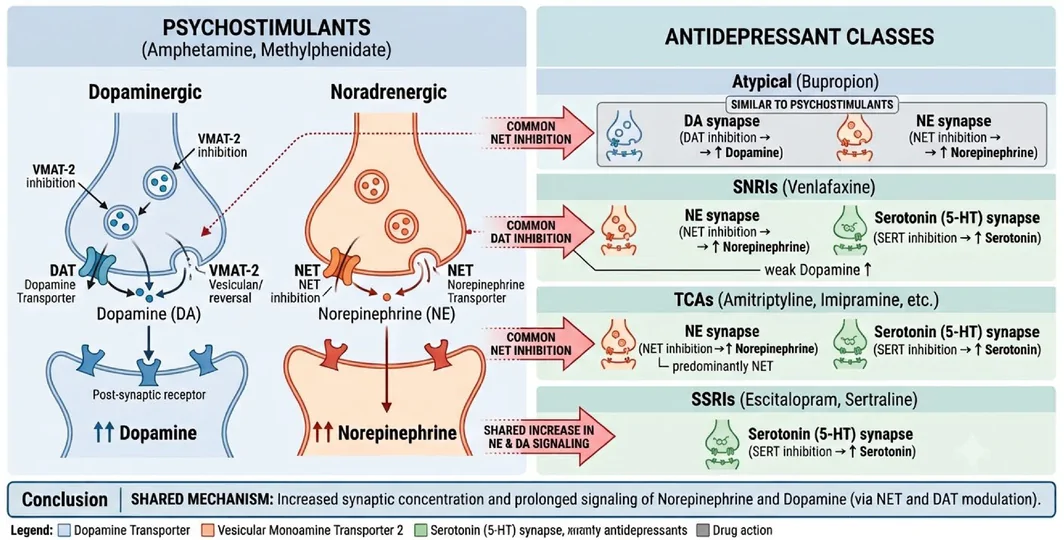
Comparison of the physiological MECHANISMS underlying dopaminergic and noradrenergic signalling induced by psychostimulants and antidepressants.

### 3.4 Pharmacokinetics of Antidepressants

Antidepressants are primarily administered orally [53] due to favourable gastrointestinal absorption, high plasma protein binding and extensive hepatic metabolism [54, 55]. In certain clinical situations, alternative routes such as intranasal or intravenous (IV) administration may be employed when rapid alleviation of depressive symptoms is required [56]. Selective serotonin reuptake inhibitors (SSRIs) generally exhibit half‐lives ranging from 15 to 26 h and are predominantly cleared via the hepatic cytochrome P450 system, which may result in clinically significant drug–drug interactions [57]. Detailed pharmacokinetic information for each antidepressant included on the ESIC monitoring list is provided in Table 2. Imipramine represents a notable exception. Following oral administration, imipramine is rapidly metabolised by CYP enzymes in the liver and intestine to its major active metabolite, desipramine. Peak plasma concentrations (t_max) are reached approximately 2 h post‐dose, regardless of dosage form. The decline in imipramine concentration is closely correlated with circulating desipramine levels; therefore, desipramine concentrations are commonly used as a surrogate marker to estimate imipramine exposure in blood [66].

**TABLE 2:** Maximum concentration (C_max_), time to maximum concentration (T_max_), half‐life (t_1/2_), area under the concentration–time curve (AUC) and apparent clearance (CL/F).

Drug	C_max_ (ng mL^−1^)	T_max_ (h)	t_1/2_ (h)	AUC	CL/F	Ref
Amitriptyline	26.0	1.0	4.9	104.5	349.1	[58]
Desipramine	21.8	4.0–12	21.0 ± 10.8	541 ± 314	108.7 ± 55.6	[59]
Nortriptyline	46.7 ± 6.3	6.6 ± 2.2	28.8 ± 6.4	1817 ± 131	1.86 ± 0.31	[60]
Bupropion	93 ± 36	1.3 ± 1	10 ± 6	469 ± 177	182 ± 68	[61]
Escitalopram	30.5 ± 12.5	5.2 ± 1.9	40.7 ± 11.3	544.3 ± 218.6	21.2 ± 8.5	[62]
Sertraline	15.1 ± 7.5	2.8	26.7 ± 5.2	198 ± 97	2.5 ± 0.9	[63, 64]
Venlafaxine	30.1 ± 17.7	6.0	8.43 ± 1.29	518 ± 462	2.824 ± 1.6	[65]

These pharmacokinetic characteristics of antidepressants are particularly relevant in the Esports context, as potential performance enhancement may be achieved by timing drug administration to coincide with peak systemic and central nervous system exposure, most commonly during the pre‐competition period [12]. From an analytical standpoint, effective anti‐doping control using LC–MS/MS [67] requires careful consideration of drug‐specific absorption rates, elimination half‐lives and metabolite formation when defining detection windows [68, 69]. In particular, the selection of appropriate screening and confirmation cut‐off concentrations should account for the expected temporal concentration profiles of parent compounds and major metabolites in the chosen biological matrix (e.g., oral fluid, urine or plasma). Failure to align sampling time and analytical sensitivity with these pharmacokinetic parameters may increase the risk of false‐negative findings, thereby limiting the ability to reliably identify antidepressant misuse in Esports competitors.

### 3.5 Side Effects and Health Risks of Misusing Antidepressants

Misuse of antidepressants, including consumption at supratherapeutic doses, use without a prescription or co‐administration with other substances, can result in severe, potentially life‐threatening and long‐term adverse health effects. A commonly reported motivation for such misuse is the pursuit of psychostimulant‐like effects. Antidepressants may be abused at high doses and through various routes of administration, including intranasal and intravenous use. The resulting adverse outcomes vary according to antidepressant class and pharmacological profile but have been reported to include seizures, cognitive impairment, confusion and psychotic‐like symptoms [70]. TCAs are associated with particularly severe consequences when misused. Their anticholinergic and antihistaminergic properties can induce confusion and delirium, while seizures represent a dose‐dependent risk. Orthostatic hypotension and falls may also occur among individuals using or misusing TCAs. Most concerning, however, are their effects on cardiac conduction. TCAs have a narrow therapeutic index and can be lethal in overdose, with cardiac arrhythmias representing the principal cause of mortality [71].

Serotonin–norepinephrine reuptake inhibitors (SNRIs) have also been implicated in misuse‐related toxicity. For example, a reported case involved a 38‐year‐old man with a history of depression and amphetamine dependence who crushed and orally ingested doses of up to 4050 mg—far exceeding the US Food and Drug Administration–recommended maximum dose of 375 mg—in an attempt to achieve an ‘amphetamine‐like high’. He subsequently presented to the emergency department with chest pain and cardiac complications [72]. Bupropion, which shares certain structural and pharmacological properties with amphetamines, has likewise been associated with significant toxicity when misused [73]. Reported cardiac effects include sinus tachycardia, while massive overdoses may result in QRS complex widening, ventricular dysrhythmias and cardiovascular collapse [73]. Nortriptyline use is associated with a range of adverse effects, including sedation, dizziness, insomnia, blurred vision, constipation and nausea. While these effects are common at antidepressant doses, they are less frequently observed at the lower doses typically used for smoking cessation [74]. In the context of Esports, even mild cognitive, psychomotor or cardiovascular side effects associated with antidepressant misuse may adversely affect fine motor coordination, reaction time, sustained attention and decision‐making, thereby undermining competitive performance and raising concerns regarding both player safety and competitive integrity.

### 3.6 Detection of Antidepressants in Biomatrices

Various researchers have previously analysed most antidepressants to monitor their presence in different biological matrices [75, 76, 77]. Blood or plasma analysis reflects recent drug use but requires invasive sampling [78]. Urine is widely employed and provides longer detection windows [79]. Saliva (oral fluid) is a non‐invasive biological matrix that is not currently included in the routine anti‐doping testing programmes of the IESF, as its anti‐doping framework aligns with the WADA code, which currently recognises urine and blood as the primary testing matrices. In contrast, the ESIC has adopted oral fluid testing [80, 81] because it provides a convenient and non‐invasive collection procedure, reduces the risk of sample adulteration, and is particularly well‐suited for detecting recent drug use [82]. Advanced analytical techniques, such as liquid chromatography–tandem mass spectrometry (LC–MS/MS), provide high specificity and sensitivity for antidepressant detection. Table 3 summarises the detection of antidepressants across different biological matrices, including limits of detection and limits of quantification.

**TABLE 3:** Summary of antidepressants detected using LC–MS/MS, with corresponding limits of detection (LOD), limits of quantification (LOQ) and sample preparation procedures.

Analyte	Biomatrix	LOD (ng mL^−1^)	LOQ (ng mL^−1^)	Sample preparation method	Ref
Amitriptyline	OF	10	10	SPE	[83]
	Urine	25	25	LLE	[77]
	Blood	2.5	2.5	SPE	[76]
Desipramine	OF	10	10	SPE	[83]
	Urine	25	25	LLE	[77]
	Blood	2.5	2.5	SPE	[76]
Imipramine	OF	10	10	SPE	[83]
	Urine	25	25	LLE	[77]
	Blood	2.5	2.5	SPE	[76]
Nortriptyline	OF	2	2	SPE	[75]
	Urine	25	25	LLE	[77]
	Blood	2.5	2.5	SPE	[76]
Bupropion	OF	10	10	SPE	[83]
	Urine	5	5	SPE	[84]
	Blood	2.5	2.5	SPE	[76]
Escitalopram	OF	10	10	SPE	[83]
	Urine	0.0038	0.01	LLE	[85]
	Blood	2.5	2.5	SPE	[76]
Sertraline	OF	10	10	SPE	[83]
	Urine	2	20	SPME	[86]
	Blood	2.5	2.5	SPE	[76]
Venlafaxine	OF	10	10	SPE	[83]
	Urine	0.24	0.79	Dilute and shoot	[87]
	Blood	2.5	2.5	SPE	[76]

Abbreviations: LLE, liquid–liquid extraction; OF, oral fluids; SPE, solid phase extraction; SPME, solid phase micro extraction.

In the fight against doping, the reliable detection of prohibited substances in biological matrices is of paramount importance [88]. Due to the unique nature of competitive Esports, the use of non‐invasive biological samples is strongly recommended for anti‐doping testing. Oral fluid is a particularly suitable matrix, as it allows sample collection without causing discomfort or compromising privacy, and without interfering with an athlete's physical performance. In contrast, invasive procedures such as blood collection may be impractical in Esports competition settings. For example, venipuncture performed immediately prior to competition could result in temporary discomfort, bruising, or reduced arm mobility [89], potentially affecting a player's ability to perform optimally when using a mouse and keyboard during gameplay. Oral fluid is composed primarily of saliva secreted by the serous and mucous cells of the salivary glands, along with additional components such as gingival crevicular fluid and lymph [90]. This clear, slightly acidic biological fluid is a complex mixture consisting predominantly of water (approximately 98%), with smaller proportions of proteins (about 0.7%), mucins (approximately 0.3%) and various electrolytes [91]. Due to its unique composition and physiological origin, oral fluid has increasingly been recognised as a valuable biological matrix for the detection of prohibited substances in forensic toxicology [92].

Oral fluid testing is widely employed for detecting drug use, particularly in contexts such as driving‐under‐the‐influence investigations and workplace drug screening programs. The method offers several advantages, including a non‐invasive and easily supervised collection procedure, reduced risk of sample adulteration and the ability to detect relatively recent drug intake. In many cases, drugs can be detected in oral fluid within approximately 48 h after administration. Furthermore, drug concentrations in oral fluid often correlate more closely with the free (pharmacologically active) fraction of drugs in plasma compared with urine testing [93, 94]. Drugs enter oral fluid primarily through passive diffusion from the bloodstream via the arterial supply to the salivary glands. Additionally, when drugs are administered through oral, smoked or intranasal routes, residues may be deposited directly in the oral cavity and subsequently detected in oral fluid samples. Consequently, both parent compounds and their metabolites can be identified using oral fluid analysis [95]. Owing to these advantages, oral fluid has been proposed by several researchers, including Miller et al. [96] and Anizan and Huestis [97], as a practical alternative to conventional biological matrices such as blood, particularly for rapid drug screening, anti‐doping testing and therapeutic drug monitoring [96, 97, 98, 99]. It also makes oral fluid a promising matrix for large‐scale testing programmes, including Esports anti‐doping, as demonstrated by Salum et al. [82], who developed and validated a UPLC–MS/MS method for the analysis of nootropic misuse in Esports using oral fluid as the biological matrix.

Analytical sensitivity was assessed using the limit of detection (LOD) and the limit of quantification (LOQ), with lower values indicating greater method sensitivity. As shown in Table 3, the LODs for the antidepressants ranged from 2 to 10 ng/mL in oral fluid.

Various sample preparation techniques have been employed, including solid‐phase extraction (SPE) [75, 87], liquid–liquid extraction (LLE) [77], solid‐phase microextraction (SPME) [100] and dilute‐and‐shoot approaches [87]. Among these, SPE is the most consistently applied method due to its superior sensitivity, selectivity, and reproducibility across different biological matrices. This performance is largely attributed to the hydrophilic–lipophilic balance (HLB) properties of the SPE sorbent, which enable efficient extraction of compounds spanning a wide range of polarities and acid–base characteristics [100]. Such versatility is particularly advantageous for multi‐residue analysis, where target analytes exhibit diverse physicochemical properties. Furthermore, SPE has been extensively validated for the simultaneous determination of pharmaceuticals and emerging contaminants [101], including drugs of abuse [102], supporting its robustness and suitability for anti‐doping applications [103]. Its ability to effectively reduce matrix interferences while enhancing analyte recovery directly improves signal‐to‐noise ratios in LC–MS/MS analysis, thereby improving LOD and LOQ [104]. In contrast, alternative techniques such as LLE offer methodological flexibility but are often limited by lower extraction efficiency, higher variability and reduced compatibility with high‐throughput workflows [105, 106]. SPME has attracted considerable attention because of its high selectivity, solvent‐free operation and simple, rapid and non‐exhaustive extraction process. These unique characteristics make it well suited for extracting a wide range of drugs from biological matrices [107]. However, SPME has several limitations when applied to the analysis of complex samples. These include relatively low extraction efficiency for highly polar compounds, strong retention of analytes on the extraction fibre due to specific analyte–adsorbent interactions and the limited availability of commercially available stationary phases, particularly for fibre‐based SPME. Collectively, these limitations restrict extraction selectivity and may reduce the applicability of SPME across a wide range of analytical applications [108, 109]. Dilute‐and‐shoot methods have been widely used for more than a decade in anti‐doping and toxicological laboratories for the analysis of a large number of substances associated with performance enhancement, such as stimulants and anabolic steroids [110, 111, 112, 113]. This approach is operationally simple, rapid and capable of detecting prohibited substances with diverse physicochemical properties in a short analytical run time [114]. Its high throughput is particularly advantageous in anti‐doping laboratories, where results are often required within 24 h before competition [115, 116]. However, because the sample undergoes minimal cleanup, dilute‐and‐shoot methods are generally associated with matrix effects, reduced analytical sensitivity and consequently lower analyte detectability, particularly for analytes present at low concentrations [113, 115]. From a critical perspective, while dilute‐and‐shoot approaches may be attractive for large‐scale screening, their susceptibility to matrix interference and limited sensitivity restrict their reliability for anti‐doping applications, where trace‐level detection is essential, particularly for prohibited substances with defined threshold values.

### 3.7 Implications for Esports

The misuse of antidepressants in Esports poses ethical, health and competitive concerns. Although these agents may offer benefits such as anxiety and panic reduction [116, 117], their use may also lead to cognitive or motor impairments that can negatively influence performance [118, 119]. In the absence of systematic monitoring, antidepressants constitute a significant blind spot in current Esports anti‐doping frameworks.

Policy interventions could include targeted awareness campaigns, strengthened medical oversight and the incorporation of antidepressant screening into routine anti‐doping procedures. Such measures should extend beyond tournaments organised solely by the ESIC to include other governing bodies, such as the International Esports Federation (IESF), a signatory to WADA. At present, IESF largely relies on WADA's Prohibited List, which has been primarily developed for traditional sports and may not adequately address substances of relevance in the Esports context [12].

Self‐medication with antidepressants is not a completely safe practice, particularly when undertaken irresponsibly. Potential risks associated with self‐medication include incorrect self‐diagnosis, delays in seeking professional medical advice, infrequent but severe adverse drug reactions, harmful drug–drug interactions, inappropriate routes of administration, incorrect dosing, unsuitable choice of therapy, masking of serious underlying conditions and an increased risk of dependence and abuse [120].

In light of these concerns, the authors emphasise the need for clinicians to exercise particular caution when prescribing antidepressants to Esports players and to consider the unique psychological stressors faced by Esports athletes. These issues raise critical questions regarding competitive fairness and the responsibility of professional governing bodies: where is the level playing field, and why are stronger regulatory controls not in place? Beyond preserving the integrity of the sport, such measures are essential to protect the long‐term health of players and the younger generations who view professional Esports athletes as role models.

Future research should adopt a staged anti‐doping approach, beginning with systematic monitoring studies to determine the prevalence of antidepressant use and misuse in Esports. Survey‐based investigations can provide population‐level data on usage patterns, timing of administration, and perceived performance effects under competitive gaming conditions. These monitoring data should then inform validation studies, including ethically approved controlled human performance trials, in which prescribed antidepressant use is evaluated against appropriate control groups to characterise cognitive, psychomotor and behavioural performance effects relevant to Esports. Finally, evidence generated from monitoring and validation phases should support enforcement‐oriented research focused on the development and analytical validation of rapid, sensitive and non‐invasive detection methods capable of reliably identifying antidepressant misuse in Esports competition settings.

## Conclusions

Esports players are exposed to multiple risk factors associated with psychological strain, including intensive training schedules, sustained competitive pressure, irregular sleep–wake cycles and limited physical activity. In this environment, the non‐medical use of antidepressants without appropriate clinical oversight or physician authorisation represents a potential risk to player health and well‐being.

While antidepressants are legitimately prescribed for the treatment of psychiatric disorders, their misuse in Esports may confer performance‐relevant effects, including enhanced emotional regulation, reduced competition‐related anxiety and improved sleep quality. Such use raises concerns aligned with established anti‐doping principles, as it may provide an unfair competitive advantage, pose health risks to players and undermine the integrity and spirit of competition. Despite these risks, antidepressant misuse remains insufficiently addressed within current Esports regulatory frameworks, with limited formal recognition beyond initiatives undertaken by the ESIC.

Accordingly, the integration of pharmacological and pharmacokinetic evidence with analytically robust detection methodologies and harmonised ethical and regulatory policies is essential to protect player health, ensure fairness, and uphold competitive integrity in Esports. In support of these objectives, the development and validation of sensitive, non‐invasive analytical approaches—particularly using oral fluid for in‐competition screening and urine for extended detection windows—are critical to enabling proportionate, evidence‐based anti‐doping measures consistent with ESIC‐aligned governance models. Looking ahead, the IESF, which adheres to the WADA code, may consider oral fluid as an alternative testing matrix for Esports because it is well suited for detecting recent drug use and provides a less invasive sampling procedure than blood. In contrast, blood collection is invasive and may temporarily impair players' comfort or hand function, which could potentially affect performance in Esports, where precise and continuous use of a mouse and keyboard is essential.

## Author Contributions

**Kassim Ali Salum:** writing – original draft, methodology, formal analysis, conceptualisation. **Shao‐Hsin Hung:** methodology, conceptualisation. **Meng‐Tsang Hsieh:** supervision, funding acquisition, conceptualisation. **Mei‐Chich Hsu:** supervision, conceptualisation. **William Chih‐Wei Chang:** writing – review and editing, supervision, funding acquisition, conceptualisation.

## Funding

This research was made possible by funding from the National Science and Technology Council, Taiwan (NSTC 114‐2410‐H‐037‐022‐MY3), and the Chi Mei Medical Center and Kaohsiung Medical University Research Foundation (114CM‐KMU‐001). The financial sources had no role in the design or execution of the study, data analysis, manuscript preparation or decision to publish.

## Conflicts of Interest

The authors declare no conflicts of interest.

## Data Availability

The authors have nothing to report.

## References

[1] T.U.o. Oklahoma , “Esports & Co‐Curricular Innovation,” Updated 7/12/2024.

[2] A. Wattanapisit , S. Wattanapisit , and S. Wongsiri , “Public Health Perspectives on Esports,” Public Health Reports 135, no. 3 (2020): 295–298. 10.1177/0033354920912718 PMC7238713 32237971

[3] B. Gaming , “The History and Evolution of Esports,” (2018).

[4] D. Tang , R. K. W. Sum , M. Li , R. Ma , P. Chung , and R. W. K. Ho , “What Is Esports? A Systematic Scoping Review and Concept Analysis of Esports,” Heliyon 9, no. 12 (2023): e23248. 10.1016/j.heliyon.2023.e23248 PMC10750068 38149186

[5] C.‐E. Niculaescu , I. Sangiorgi , and A. R. Bell , “Venture Capital Financing in the Esports Industry,” Research in International Business and Finance 65 (2023): 101951.

[6] F. B. Schuch , R. A. Bulzing , J. Meyer , et al., “Associations of Moderate to Vigorous Physical Activity and Sedentary Behavior With Depressive and Anxiety Symptoms in Self‐Isolating People During the COVID‐19 Pandemic: A Cross‐Sectional Survey in Brazil,” Psychiatry Research 292 (2020): 113339. 10.1016/j.psychres.2020.113339 PMC7384423 32745795

[7] T. Li , Mental Health Concerns in E‐Sport Athletes: Lessons From a Legend (American Psychiatric Association, 2024).

[8] D. Poulus , T. J. Coulter , M. G. Trotter , and R. Polman , “Stress and Coping in Esports and the Influence of Mental Toughness,” Frontiers in Psychology 11 (2020): 628. 10.3389/fpsyg.2020.00628 PMC7191198 32390900

[9] H. J. Hong , “Esports: The Need for a Structured Support System for Players,” European Sport Management Quarterly 23, no. 5 (2023): 1430–1453.

[10] ESIC , “ESIC Guidance on International Esports Federation (IESF) WADA Anti Doping Rule Violation Against Arslan Ash,” (2025), https://esic.gg/press‐release/esic‐guidance‐on‐iesf‐wada‐anti‐doping‐rule‐violation‐on‐arslan‐ash/.

[11] ESIC , “ESIC Prohibited Substance List,” (2024), https://esic.gg/codes/esic‐prohibited‐list/.

[12] K. A. Salum , S. H. Hung , M. T. Hsieh , and W. C. W. Chang , “Doping in Esports: A Review of Nootropics and Analytical Methods for Their Detection,” Physical Education 57, no. 4 (2025): 1–22.

[13] E. J. Ip , E. P. T. Urbano , J. Caballero , et al., “The Video Gamer 500: Performance‐Enhancing Drug Use and Internet Gaming Disorder Among Adult Video Gamers,” Computers in Human Behavior 123 (2021): 106890.

[14] T. Schöber and G. Stadtmann , “The Dark Side of E‐Sports—An Analysis of Cheating, Doping & Match‐Fixing Activities and Their Countermeasures,” International Journal of Esports 1, no. 1 (2022), https://www.ijesports.org/article/98/html.

[15] Y. Feng , M. Zheng , X. Zhang , et al., “Analysis of Four Antidepressants in Plasma and Urine by Gas Chromatography‐Mass Spectrometry Combined With Sensitive and Selective Derivatization,” Journal of Chromatography A 1600 (2019): 33–40. 10.1016/j.chroma.2019.04.038 31040033

[16] T. Rosado , A. Gonçalves , A. Martinho , et al., “Simultaneous Quantification of Antidepressants and Metabolites in Urine and Plasma Samples by GC–MS for Therapeutic Drug Monitoring,” Chromatographia 80, no. 2 (2017): 301–328.

[17] E. Zilfidou , A. Kabir , K. G. Furton , and V. Samanidou , “An Improved Fabric Phase Sorptive Extraction Method for the Determination of Five Selected Antidepressant Drug Residues in Human Blood Serum Prior to High Performance Liquid Chromatography With Diode Array Detection,” Journal of Chromatography B 1125 (2019): 121720. 10.1016/j.jchromb.2019.121720 31326644

[18] R. S. Silva , Esports and Cheating: A Challenge to Competitive Gaming (Medium, 2023).

[19] C. Hamstead , “‘Nobody Talks About It Because Everyone Is on It’: Adderall Presents Esports With an Enigma,” (2020), https://www.washingtonpost.com/video‐games/Esports/2020/02/13/Esports‐adderall‐drugs/.

[20] S. K. Park , I. Y. Chang , and S. Y. Roh , “Is Esports Threatened by e‐Doping?: Focusing on the Perceptions of Professional Esports Players and Coaches in South Korea,” Sport in Society (2025): 1–24.

[21] P. Bell , C. Ten Have , and M. Lauchs , “A Case Study Analysis of a Sophisticated Sports Doping Network: Lance Armstrong and the USPS Team,” International Journal of Law, Crime and Justice 46 (2016): 57–68.

[22] W. J. a. M. A. de Brown and M. A. de Matviuk , “Sports Celebrities and Public Health: Diego Maradona's Influence on Drug Use Prevention,” Journal of Health Communication 15, no. 4 (2010): 358–373. 10.1080/10810730903460575 20574875

[23] A. Arduini and V. A. Zammit , “A Tennis Lesson: Sharp Practice in the Science Behind the Sharapova Case,” Postgraduate Medical Journal 92, no. 1090 (2016): 429–430. 10.1136/postgradmedj-2016-134124 PMC4975811 27252310

[24] A. Richardson , “Entering Cheat Codes or to Play True: Where Is Anti‐Doping Going Within Esports?,” International Journal of Esports 1, no. 1 (2024), https://www.ijesports.org/article/144/html.

[25] WADA , “The Prohibited List,” (2026).

[26] N. B. Allen , “Book Review: Cognitive Therapy of Depression,” Australian and New Zealand Journal of Psychiatry 36, no. 2 (2002): 275–278. 10.1046/j.1440-1614.2002.t01-5-01015.x 11982561

[27] D. H. Han and P. F. Renshaw , “Bupropion in the Treatment of Problematic Online Game Play in Patients With Major Depressive Disorder,” Journal of Psychopharmacology 26, no. 5 (2012): 689–696. 10.1177/0269881111400647 PMC4638175 21447539

[28] A. Richardson , “An Updated Classification of Cheating in Esports,” International Journal of Esports 1, no. 1 (2025), https://www.ijesports.org/article/149/html.

[29] H. S. Arvinder‐Singh , R. Philip , and A. Winson , “A New Enigma: Doping in E‐Sports,” Medical Journal of Malaysia 79, no. 1 (2024): 111–112. 38287766

[30] G. Aonso‐Diego , A. González‐Roz , S. Weidberg , and R. Secades‐Villa , “Depression, Anxiety, and Stress in Young Adult Gamers and Their Relationship With Addictive Behaviors: A Latent Profile Analysis,” Journal of Affective Disorders 366 (2024): 254–261. 10.1016/j.jad.2024.08.203 39218313

[31] IESF , “Prohibited Substances,” https://iesf.org/anti‐doping/prohibited‐substances.

[32] P. Hodgkins , M. Shaw , D. Coghill , and L. Hechtman , “Amfetamine and Methylphenidate Medications for Attention‐Deficit/Hyperactivity Disorder: Complementary Treatment Options,” European Child & Adolescent Psychiatry 21, no. 9 (2012): 477–492. 10.1007/s00787-012-0286-5 PMC3432777 22763750

[33] S. V. Faraone , “The Pharmacology of Amphetamine and Methylphenidate: Relevance to the Neurobiology of Attention‐Deficit/Hyperactivity Disorder and Other Psychiatric Comorbidities,” Neuroscience & Biobehavioral Reviews 87 (2018): 255–270. 10.1016/j.neubiorev.2018.02.001 PMC8063758 29428394

[34] C. A. Roberts , A. Jones , H. Sumnall , S. H. Gage , and C. Montgomery , “How Effective Are Pharmaceuticals for Cognitive Enhancement in Healthy Adults? A Series of Meta‐Analyses of Cognitive Performance During Acute Administration of Modafinil, Methylphenidate and D‐Amphetamine,” European Neuropsychopharmacology 38 (2020): 40–62. 10.1016/j.euroneuro.2020.07.002 32709551

[35] K. Vo , P. J. Neafsey , and C. A. Lin , “Concurrent Use of Amphetamine Stimulants and Antidepressants by Undergraduate Students,” Patient Preference and Adherence 9 (2015): 161–172. 10.2147/PPA.S74602 PMC4309786 25653508

[36] B. Pires , L. M. Rosendo , A. T. Brinca , et al., “The Therapeutic Potential of Amphetamine‐Like Psychostimulants,” Life (Basel) 13, no. 11 (2023): 2180. 10.3390/life13112180 PMC10671856 38004320

[37] T. Favrod‐Coune and B. Broers , “The Health Effect of Psychostimulants: A Literature Review,” Pharmaceuticals (Basel) 3, no. 7 (2010): 2333–2361. 10.3390/ph3072333 PMC4036656 27713356

[38] T. M. Hillhouse and J. H. Porter , “A Brief History of the Development of Antidepressant Drugs: From Monoamines to Glutamate,” Experimental and Clinical Psychopharmacology 23, no. 1 (2015): 1–21. 10.1037/a0038550 PMC4428540 25643025

[39] P. S. D'Aquila , M. Collu , G. L. Gessa , and G. Serra , “The Role of Dopamine in the Mechanism of Action of Antidepressant Drugs,” European Journal of Pharmacology 405, no. 1 (2000): 365–373. 10.1016/s0014-2999(00)00566-5 11033341

[40] J. R. Docherty and H. A. Alsufyani , “Pharmacology of Drugs Used as Stimulants,” Journal of Clinical Pharmacology 61, no. S2 (2021): S53–S69. 10.1002/jcph.1918 34396557

[41] J. S. M. A. R. A Saadabadi , “Desipramine,” (2025).

[42] E. W. McClure and R. N. Daniels , “Classics in Chemical Neuroscience: Amitriptyline,” ACS Chemical Neuroscience 12, no. 3 (2021): 354–362. 10.1021/acschemneuro.0c00467 33438398

[43] Y. Bohren , L. H. Tessier , S. Megat , et al., “Antidepressants Suppress Neuropathic Pain by a Peripheral β2‐Adrenoceptor Mediated Anti‐TNFα Mechanism,” Neurobiology of Disease 60 (2013): 39–50. 10.1016/j.nbd.2013.08.012 23978467

[44] G. M. J. R. G. S. A. H. A. Saadabadi , “Nortriptyline,” (2023), https://www.ncbi.nlm.nih.gov/books/NBK482214/.

[45] M. P. M. Boks , “Chapter 23—Epigenetic Effects of Currently Used Psychotropic Drugs,” in Epigenetics in Psychiatry, eds. J. Peedicayil , D. R. Grayson , and D. Avramopoulos (Academic Press, 2014), 481–496.

[46] H. Zhong , N. Haddjeri , and C. Sánchez , “Escitalopram, an Antidepressant With an Allosteric Effect at the Serotonin Transporter—A Review of Current Understanding of Its Mechanism of Action,” Psychopharmacology 219, no. 1 (2012): 1–13. 10.1007/s00213-011-2463-5 21901317

[47] C. Costagliola , F. Parmeggiani , F. Semeraro , and A. Sebastiani , “Selective Serotonin Reuptake Inhibitors: A Review of Its Effects on Intraocular Pressure,” Current Neuropharmacology 6, no. 4 (2008): 293–310. 10.2174/157015908787386104 PMC2701282 19587851

[48] F. P. Bymaster , L. J. Dreshfield‐Ahmad , P. G. Threlkeld , et al., “Comparative Affinity of Duloxetine and Venlafaxine for Serotonin and Norepinephrine Transporters In Vitro and In Vivo, Human Serotonin Receptor Subtypes, and Other Neuronal Receptors,” Neuropsychopharmacology 25, no. 6 (2001): 871–880. 10.1016/S0893-133X(01)00298-6 11750180

[49] R. A. Sansone and L. A. Sansone , “Serotonin Norepinephrine Reuptake Inhibitors: A Pharmacological Comparison,” Innovations in Clinical Neuroscience 11, no. 3–4 (2014): 37–42. PMC4008300 24800132

[50] L. Dean and M. Kane , “Venlafaxine Therapy and CYP2D6 Genotype,” Medical Genetics Summaries, (2015), https://www.ncbi.nlm.nih.gov/books/.

[51] G. Canıtez , R. Şimşek , D. Arca Çakır , H. Tezel Yalçın , T. Baydar , and P. Erkekoğlu , “Toxicity of Serotonin‐Norepinephrine Reuptake Inhibitors (SNRIs) During Pregnancy and Lactation,” İstanbul Journal of Pharmacy 54, no. 2 (2024): 255–267.

[52] L. P. Dwoskin , A. S. Rauhut , K. A. King‐Pospisil , and M. T. Bardo , “Review of the Pharmacology and Clinical Profile of Bupropion, an Antidepressant and Tobacco Use Cessation Agent,” CNS Drug Reviews 12, no. 3–4 (2006): 178–207. 10.1111/j.1527-3458.2006.00178.x PMC6506196 17227286

[53] A. Das , C. C. Obiozor , D. Elwadhi , and M. A. Fuller , “Options When Anti‐Depressants Cannot Be Used in Conventional Ways. Clinical Case and Review of Literature,” Personalized Medicine in Psychiatry 15‐16 (2019): 22–27.

[54] S. Bashiardes and C. Christodoulou , “Orally Administered Drugs and Their Complicated Relationship With Our Gastrointestinal Tract,” Microorganisms 12, no. 2 (2024): 242. 10.3390/microorganisms12020242 PMC10893523 38399646

[55] Z. Vinarov , M. Abdallah , J. A. G. Agundez , et al., “Impact of Gastrointestinal Tract Variability on Oral Drug Absorption and Pharmacokinetics: An UNGAP Review,” European Journal of Pharmaceutical Sciences 162 (2021): 105812. 10.1016/j.ejps.2021.105812 33753215

[56] J. An , X. Li , Z. Li , J. Wang , and Y. Pan , “Intranasal Administration in Modulating Depressive‐Like Behavior and Reconstructing Treatment Paradigms Through Neuroinflammation and Neurotrophic Pathways,” Journal of Nanobiotechnology 24, no. 1 (2026): 463. 10.1186/s12951-026-04417-y PMC13200309 41987143

[57] C. L. DeVane , “Pharmacokinetics of the Newer Antidepressants: Clinical Relevance,” American Journal of Medicine 97, no. 6 (1994): S13–S23. 10.1016/0002-9343(94)90359-x 7992822

[58] W. A. Garland , B. H. Min , and D. J. Birkett , “The Kinetics of Amitriptyline Following Single Oral Dose Administration to Man,” Research Communications in Chemical Pathology and Pharmacology 3 (1978): 475–484. 734227

[59] R. Z. Harris , M. Salfi , E. Posvar , D. Hoelscher , and D. Padhi , “Pharmacokinetics of Desipramine HCl When Administered With Cinacalcet HCl,” European Journal of Clinical Pharmacology 63, no. 2 (2007): 159–163. 10.1007/s00228-006-0129-8 16680561

[60] Q.‐Y. Yue , Z. H. Zhong , G. Tybring , et al., “Pharmacokinetics of Nortriptyline and Its 10‐Hydroxy Metabolite in Chinese Subjects of Different CYP2D6 Genotypes,” Clinical Pharmacology & Therapeutics 64, no. 4 (1998): 384–390. 10.1016/S0009-9236(98)90069-8 9797795

[61] J. N. Connarn , S. Flowers , M. Kelly , et al., “Pharmacokinetics and Pharmacogenomics of Bupropion in Three Different Formulations With Different Release Kinetics in Healthy Human Volunteers,” AAPS Journal 19, no. 5 (2017): 1513–1522. 10.1208/s12248-017-0102-8 28685396

[62] A. Periclou , N. Rao , T. Sherman , D. Ventura , and W. Abramowitz , Single‐Dose Pharmacokinetic Study of Escitalopram in Adolescents and Adults (Pharmacotherapy Publications Inc., 2003), 1361–1362.

[63] J. M. Cooper , S. B. Duffull , A. S. Saiao , and G. K. Isbister , “The Pharmacokinetics of Sertraline in Overdose and the Effect of Activated Charcoal,” British Journal of Clinical Pharmacology 79, no. 2 (2015): 307–315. 10.1111/bcp.12500 PMC4309636 25155462

[64] D. A. Axelson , et al., “Sertraline Pharmacokinetics and Dynamics in Adolescents,” Journal of the American Academy of Child & Adolescent Psychiatry 41, no. 9 (2002): 1037–1044. 10.1097/00004583-200209000-00003 12218424

[65] A. I. Nichols , K. Focht , Q. Jiang , S. H. Preskorn , and C. P. Kane , “Pharmacokinetics of Venlafaxine Extended Release 75 mg and Desvenlafaxine 50 mg in Healthy CYP2D6 Extensive and Poor Metabolizers: A Randomized, Open‐Label, Two‐Period, Parallel‐Group, Crossover Study,” Clinical Drug Investigation 31, no. 3 (2011): 155–167. 10.2165/11586630-000000000-00000 21288052

[66] Y. Cho Hsu , S. Chang Hsiao , H. Wen Huang , P. Yang Ho , C. Yi Lin , and T. Chun Chuang , “Population Pharmacokinetic Study of Imipramine in Taiwan People Healthy Volunteers,” American Journal of BioMedicine 12, no. 1 (2024): 21–32.

[67] M. Thevis , A. Thomas , and W. Schänzer , “Current Role of LC‐MS(/MS) in Doping Control,” Analytical and Bioanalytical Chemistry 401, no. 2 (2011): 405–420. 10.1007/s00216-011-4859-9 21424519

[68] M. Thevis , “Sports Drug Testing,” in Karch's Drug Abuse Handbook, ed. B. A. G. S. B. Karch (Taylor & Francis Group, 2022), 369–436.

[69] J. Breuer , A. Thomas , P. Delahaut , W. Schänzer , H. Geyer , and M. Thevis , “Investigations Into the Concentration and Metabolite Profiles of Stanozolol and LGD‐4033 in Blood Plasma and Seminal Fluid Using Liquid Chromatography High‐Resolution Mass Spectrometry,” Analytical and Bioanalytical Chemistry 415, no. 4 (2023): 669–681. 10.1007/s00216-022-04456-y PMC9839828 36441233

[70] E. A. Evans and M. A. Sullivan , “Abuse and Misuse of Antidepressants,” Substance Abuse and Rehabilitation 5, no. null (2014): 107–120. 10.2147/SAR.S37917 PMC4140701 25187753

[71] A. F. Schatzberg and C. B. Nemeroff , Essentials of Clinical Psychopharmacology, 3rd ed. (American Psychiatric Pub, 2013).

[72] Group, W.S.M.W , Minimum Required Performance Levels and Applicable Minimum Reporting Levels for Non‐THRESHOLD Substances Analyzed by Chromatographic ‐ Mass Spectrometric Analytical Methods (WADA, 2022).

[73] N. Stall , J. Godwin , and D. D. Juurlink , “Bupropion Abuse and Overdose,” CMAJ 186, no. 13 (2014): 1015–1015. 10.1503/cmaj.131534 PMC4162783 24778361

[74] J. R. Hughes , L. F. Stead , and T. Lancaster , “Nortriptyline for Smoking Cessation: A Review,” Nicotine & Tobacco Research 7, no. 4 (2005): 491–499. 10.1080/14622200500185298 16085520

[75] A. de Castro , M. Concheiro , O. Quintela , A. Cruz , and M. López‐Rivadulla , “LC–MS/MS Method for the Determination of Nine Antidepressants and Some of Their Main Metabolites in Oral Fluid and Plasma: Study of Correlation Between Venlafaxine Concentrations in Both Matrices,” Journal of Pharmaceutical and Biomedical Analysis 48, no. 1 (2008): 183–193. 10.1016/j.jpba.2008.05.024 18602787

[76] L. Kim , “Development and Validation of a LC‐MS/MS Method for Detection and Quantification of 18 Antidepressants in Whole Blood,” (2019).

[77] J. L. Poklis , C. E. Wolf , A. Goldstein , M. L. Wolfe , and A. Poklis , “Detection and Quantification of Tricyclic Antidepressants and Other Psychoactive Drugs in Urine by HPLC/MS/MS for Pain Management Compliance Testing,” Journal of Clinical Laboratory Analysis 26, no. 4 (2012): 286–294. 10.1002/jcla.21519 PMC3969737 22811363

[78] B. M. Kapur and K. Aleksa , “What the Lab Can and Cannot Do: Clinical Interpretation of Drug Testing Results,” Critical Reviews in Clinical Laboratory Sciences 57, no. 8 (2020): 548–585. 10.1080/10408363.2020.1774493 32609540

[79] S. E. Hadland and S. Levy , “Objective Testing: Urine and Other Drug Tests,” Child and Adolescent Psychiatric Clinics of North America 25, no. 3 (2016): 549–565. 10.1016/j.chc.2016.02.005 PMC4920965 27338974

[80] N. Williams , “Chapter 12: Doping and Other Illicit Performance‐Enhancing Practices in Esports,” in Esports Law and Practice (Edward Elgar Publishing, 2025), 268–293.

[81] virtus.pro , “Doping‐Test in Esports. Watch How NEO Goes Through It on ESL ONE New York 2017,” (2017).

[82] K. A. Salum , M. T. Hsieh , S. H. Hung , H. C. Chen , and W. C. W. Chang , “Development and Validation of UPLC–MS/MS Method for Detecting Nootropic Misuse in Oral Fluids: A Step Towards Combating Doping in Esports,” Analytica Chimica Acta 1383 (2026): 344871. 10.1016/j.aca.2025.344871 41352917

[83] S. S. Shin , D. Borg , and R. Stripp , “Developing and Validating a Fast and Accurate Method to Quantify 18 Antidepressants in Oral Fluid Samples Using SPE and LC–MS‐MS,” Journal of Analytical Toxicology 44, no. 6 (2020): 610–617. 10.1093/jat/bkz117 32115632

[84] R. Coles and E. D. Kharasch , “Stereoselective Analysis of Bupropion and Hydroxybupropion in Human Plasma and Urine by LC/MS/MS,” Journal of Chromatography B 857, no. 1 (2007): 67–75. 10.1016/j.jchromb.2007.07.007 17656162

[85] W. F. S. Badulla , Z. Atkoşar , G. Arli , and E. Şener , “Application of LC–ESI‐MS/MS Method for Analysis of Escitalopram Oxalate in Human Urine and Pharmaceutical Dosage Forms,” Journal of Chromatographic Science 58, no. 2 (2019): 91–97. 10.1093/chromsci/bmz073 31681957

[86] E. V. S. Maciel , A. L. . Toffoli , J. . S. Alves , and F. M. Lanças , “Multidimensional Liquid Chromatography Employing a Graphene Oxide Capillary Column as the First Dimension: Determination of Antidepressant and Antiepileptic Drugs in Urine,” Molecules 25, no. 5 (2020): 1092. 10.3390/molecules25051092 PMC7179208 32121435

[87] D. Florou , A. Orfanidis , and V. A. Boumba , “Development and Validation of a ‘Dilute and Shoot’ LC–MS/MS Method in Urine Suitable for Screening and Confirmation Analyses in a Clinical Setting,” Journal of Chromatography B 1265 (2025): 124750. 10.1016/j.jchromb.2025.124750 40795634

[88] R. Faiss , J. Saugy , and M. Saugy , “Fighting Doping in Elite Sports: Blood for All Tests!,” Frontiers in Sports and Active Living 1 (2019): 30. 10.3389/fspor.2019.00030 PMC7739585 33344954

[89] I. J. Kim , H. Cho , M. S. Yi , et al., “Diagnosis and Treatment of Nerve Injury Following Venipuncture—A Report of Two Cases,” Anesthesia and Pain Medicine (Seoul) 16, no. 3 (2021): 299–304. 10.17085/apm.21010 PMC8342828 34289298

[90] A. Di Trana , D. Berardinelli , A. Tini , and S. Zaami , “The Targeted Analysis of New Psychoactive Substances in Oral Fluid Through Chromatographic‐Spectrometric Methods: Review of Recent Findings,” European Review for Medical and Pharmacological Sciences 26, no. 3 (2022): 750–754. 10.26355/eurrev_202202_27982 35179740

[91] S. P. Humphrey and R. T. Williamson , “A Review of Saliva: Normal Composition, Flow, and Function,” Journal of Prosthetic Dentistry 85, no. 2 (2001): 162–169. 10.1067/mpr.2001.113778 11208206

[92] E. G. de Campos , B. R. B. da Costa , F. S. dos Santos , et al., “Alternative Matrices in Forensic Toxicology: A Critical Review,” Forensic Toxicology 40, no. 1 (2022): 1–18. 10.1007/s11419-021-00596-5 PMC9715501 36454488

[93] C. Moore , “Oral Fluid and Hair in Workplace Drug Testing Programs: New Technology for Immunoassays,” Drug Testing and Analysis 3, no. 3 (2011): 166–168. 10.1002/dta.140 21425472

[94] A. G. Verstraete , “Detection Times of Drugs of Abuse in Blood, Urine, and Oral Fluid,” Therapeutic Drug Monitoring 26, no. 2 (2004): 200–205. 10.1097/00007691-200404000-00020 15228165

[95] E. J. Cone and M. A. Huestis , “Interpretation of Oral Fluid Tests for Drugs of Abuse,” Annals of the New York Academy of Sciences 1098 (2007): 51–103. 10.1196/annals.1384.037 PMC2700061 17332074

[96] G. D. Miller , R. M. van Wagoner , B. J. Bruno , J. D. Husk , M. N. Fedoruk , and D. Eichner , “Investigating Oral Fluid and Exhaled Breath as Alternative Matrices for Anti‐Doping Testing: Analysis of 521 Matched Samples,” Journal of Pharmaceutical and Biomedical Analysis 176 (2019): 112810. 10.1016/j.jpba.2019.112810 31430626

[97] S. Anizan and M. A. Huestis , “The Potential Role of Oral Fluid in Antidoping Testing,” Clinical Chemistry 60, no. 2 (2014): 307–322. 10.1373/clinchem.2013.209676 PMC4545493 24153253

[98] M. Resztak , A. Czyrski , and J. Sobiak , “Saliva as a Matrix for Therapeutic Drug Monitoring and Disease Biomarkers in Children and Adolescents,” Pharmacological Reports 77, no. 4 (2025): 921–961. 10.1007/s43440-025-00732-7 PMC12241277 40358859

[99] F. Kunkel , E. Fey , D. Borg , R. Stripp , and C. Getto , “Assessment of the Use of Oral Fluid as a Matrix for Drug Monitoring in Patients Undergoing Treatment for Opioid Addiction,” Journal of Opioid Management 11, no. 5 (2015): 435–442. 10.5055/jom.2015.0293 26535971

[100] J. J. Grandy , V. Singh , M. Lashgari , M. Gauthier , and J. Pawliszyn , “Development of a Hydrophilic Lipophilic Balanced Thin Film Solid Phase Microextraction Device for Balanced Determination of Volatile Organic Compounds,” Analytical Chemistry 90, no. 23 (2018): 14072–14080. 10.1021/acs.analchem.8b04544 30372047

[101] M. Gros , M. Petrović , and D. Barceló , “Development of a Multi‐Residue Analytical Methodology Based on Liquid Chromatography–Tandem Mass Spectrometry (LC–MS/MS) for Screening and Trace Level Determination of Pharmaceuticals in Surface and Wastewaters,” Talanta 70, no. 4 (2006): 678–690. 10.1016/j.talanta.2006.05.024 18970827

[102] M. R. Boleda , M. T. Galceran , and F. Ventura , “Trace Determination of Cannabinoids and Opiates in Wastewater and Surface Waters by Ultra‐Performance Liquid Chromatography–Tandem Mass Spectrometry,” Journal of Chromatography A 1175, no. 1 (2007): 38–48. 10.1016/j.chroma.2007.10.029 17980892

[103] M. Kolmonen , A. Leinonen , A. Pelander , and I. Ojanperä , “A General Screening Method for Doping Agents in Human Urine by Solid Phase Extraction and Liquid Chromatography/Time‐of‐Flight Mass Spectrometry,” Analytica Chimica Acta 585, no. 1 (2007): 94–102. 10.1016/j.aca.2006.12.028 17386652

[104] M. Weerasooriyagedara , J. M. Partington , W. Alghamdi , M. L. Akele , S. Freguia , and B. O. Clarke , “Development and Validation of a Multi‐Class Solid‐Phase Extraction and Liquid Chromatography Tandem Mass Spectrometry Method for Emerging Contaminants in Water,” Journal of Chromatography A 1768 (2026): 466655. 10.1016/j.chroma.2025.466655 41506022

[105] M. P. Juhascik and A. J. Jenkins , “Comparison of Liquid/Liquid and Solid‐Phase Extraction for Alkaline Drugs,” Journal of Chromatographic Science 47, no. 7 (2009): 553–557. 10.1093/chromsci/47.7.553 19772726

[106] W. R. Barrionuevo and F. M. Lanças , “Comparison of Liquid–Liquid Extraction (LLE), Solid‐Phase Extraction (SPE), and Solid‐Phase Microextraction (SPME) for Pyrethroid Pesticides Analysis From Enriched River Water,” Bulletin of Environmental Contamination and Toxicology 69, no. 1 (2002): 123–128. 10.1007/s00128-002-0018-5 12053266

[107] D. Leszczyńska , A. Hallmann , N. Treder , T. Bączek , and A. Roszkowska , “Recent Advances in the Use of SPME for Drug Analysis in Clinical, Toxicological, and Forensic Medicine Studies,” Talanta 270 (2024): 125613. 10.1016/j.talanta.2023.125613 38159351

[108] K. Delińska , P. W. Rakowska , and A. Kloskowski , “Porous Material‐Based Sorbent Coatings in Solid‐Phase Microextraction Technique: Recent Trends and Future Perspectives,” TrAC Trends in Analytical Chemistry 143 (2021): 116386.

[109] M. W. J. van Hout , et al., “Chapter 1—New Developments in Integrated Sample Preparation for Bioanalysis,” in Handbook of Analytical Separations, ed. I. D. Wilson (Elsevier Science B.V., 2003), 1–44.

[110] E. F. Göktaş , E. Kabil , E. Söylemez Yeşilçimen , and L. Dirikolu , “Screening Analysis of Doping Agents in Horse Urine and Plasma With Dilute and Shoot Using Liquid Chromatography High Resolution Mass Spectrometry,” Analyst 150, no. 4 (2025): 773–792. 10.1039/d4an01501k 39846867

[111] Y. Dong , K. Yan , Y. Ma , et al., “A Sensitive Dilute‐and‐Shoot Approach for the Simultaneous Screening of 71 Stimulants and 7 Metabolites in Human Urine by LC–MS‐MS With Dynamic MRM,” Journal of Chromatographic Science 53, no. 9 (2015): 1528–1535. 10.1093/chromsci/bmv048 25953781

[112] D. Oliveira , A. de Araújo , W. Ribeiro , et al., “Screening Method of Mildronate and Over 300 Doping Agents by Reversed‐Phase Liquid Chromatography‐High Resolution Mass Spectrometry,” Journal of Pharmaceutical and Biomedical Analysis 195 (2021): 113870. 10.1016/j.jpba.2020.113870 33453569

[113] K. Tamama , “Dilute and Shoot Approach for Toxicology Testing,” Frontiers in Chemistry 11 (2023): 1278313. 10.3389/fchem.2023.1278313 PMC10749341 38146427

[114] K. Aarika , R. Rajyalakshmi , L. V. Nalla , and S. N. R. Gajula , “From Complexity to Clarity: Expanding Metabolome Coverage With Innovative Analytical Strategies,” Journal of Separation Science 48, no. 2 (2025): e70099. 10.1002/jssc.70099 PMC11836935 39968702

[115] K. Deventer , O. J. Pozo , A. G. Verstraete , and P. van Eenoo , “Dilute‐and‐Shoot‐Liquid Chromatography‐Mass Spectrometry for Urine Analysis in Doping Control and Analytical Toxicology,” TrAC Trends in Analytical Chemistry 55 (2014): 1–13.

[116] F. Badoud , D. Guillarme , J. Boccard , et al., “Analytical Aspects in Doping Control: Challenges and Perspectives,” Forensic Science International 213, no. 1 (2011): 49–61. 10.1016/j.forsciint.2011.07.024 21824736

[117] W. Andrews , G. Parker , and E. Barrett , “The SSRI Antidepressants: Exploring Their “Other” Possible Properties,” Journal of Affective Disorders 49, no. 2 (1998): 141–144. 10.1016/s0165-0327(97)00203-6 9609678

[118] I. Hindmarch , “The Effects of Antidepressants on Psychomotor Function With Particular Reference to Reboxetine,” European Neuropsychopharmacology 7, no. 1, Supplement 1 (1997): S17–S21. 10.1016/s0924-977x(97)00416-1 9169307

[119] J. S. Saczynski , A. B. Rosen , R. J. McCammon , et al., “Antidepressant Use and Cognitive Decline: The Health and Retirement Study,” American Journal of Medicine 128, no. 7 (2015): 739–746. 10.1016/j.amjmed.2015.01.007 PMC4618694 25644319

[120] R. Maria Esperanza , “Risks of Self‐Medication Practices,” Current Drug Safety 5, no. 4 (2010): 315–323. 10.2174/157488610792245966 20615179

